# Stereotactic Ablative Radiation Therapy for Sacral Bone Metastasis in Recurrent Type A Thymoma: A Two-Year Follow-Up Demonstrating Pain Reduction and Local Control

**DOI:** 10.7759/cureus.67142

**Published:** 2024-08-18

**Authors:** Atsuto Katano, Daichi Sugahara, Ayane Yasui, Yuki Nozawa, Hideomi Yamashita

**Affiliations:** 1 Radiology, The University of Tokyo Hospital, Tokyo, JPN; 2 Radiation Oncology, The University of Tokyo Hospital, Tokyo, JPN

**Keywords:** pain, recurrence, thymus neoplasms, radiotherapy, thymoma

## Abstract

A 74-year-old Asian man presented with sacral bone metastasis-related pain caused by a metastatic thymoma. Computed tomography revealed an approximately 6-cm sacral mass, which was confirmed as a metastatic thymoma. The patient was referred to our department and underwent stereotactic ablative radiation therapy (SABR) using volumetric modulated arc therapy and received a total dose of 35 Gy in five fractions. One year after SABR, the sacral lesion had decreased in size, and the pain medication was reduced. After two years, the patient no longer required pain medication, indicating successful management of bone metastases in recurrent Type A Thymoma.

## Introduction

The thymus is essential for the development of the immune system, where it regulates the maturation, selection, and migration of T lymphocytes [1]. Thymoma is a rare neoplasm with a higher incidence among Asians/Pacific Islanders and Black individuals than among White individuals [2]. One-third of the patients are asymptomatic, and the condition is specifically associated with autoimmune diseases, such as myasthenia gravis [3]. Complete surgical resection is the mainstay of thymoma therapy [4]. Locally advanced thymoma and thymic carcinoma necessitate adjuvant therapies, such as chemotherapy and radiation therapy, to reduce recurrence risk and enhance survival outcomes [5]. If recurrence occurs, surgical resection is the preferred salvage treatment [6].

Stereotactic ablative radiation therapy (SABR) is a promising treatment for localized lesions. It allows the precise delivery of high-dose radiation to the tumor while minimizing damage to the surrounding healthy tissues [7]. This approach is particularly beneficial for patients who are not candidates for surgery or conventional radiation therapy because of comorbidities or tumor location.

In this report, we present the case of a patient with Masaoka stage III type A thymoma who was treated with SABR. The patient initially underwent surgical resection of the primary thymoma. Recurrence was detected in the sacral bone after two years, which is a rare and challenging site for thymoma metastasis. Margaritora et al. reported that out of 315 patients who underwent resection with radical intent for thymoma, 43 experienced a relapse. Among these 43 patients, the recurrences were predominantly found in the pleura, mediastinum, and lungs, with only one case of relapse occurring in the bone [8]. Herein, we discuss the clinical outcomes of SABR for the management of this rare and complex condition.

## Case presentation

A 74-year-old Asian man was referred to our department for the management of pain due to sacral bone metastasis. He experienced pain and was prescribed one tablet (100 mg) of celecoxib twice daily, one tablet (2.5 mg) of mirogabalin twice daily, and three orally disintegrating tramadol hydrochloride tablets (25 mg/tablet) four times daily for pain management.

**Figure 1:**
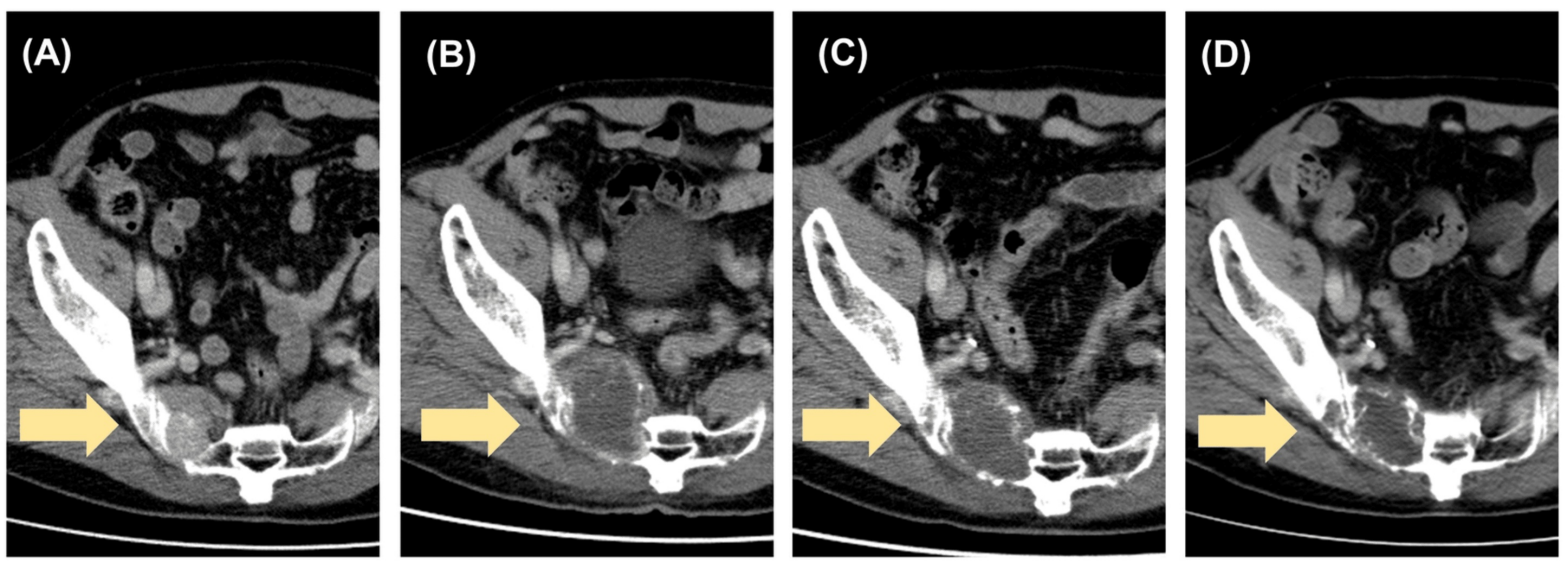
Case of a 74-year-old man with sacral bone metastasis from thymoma The yellow arrows indicated the sacral bone metastasis from thymoma. (A) A computed tomography scan showing a 3-cm mass in the sacral bone at the time of recurrence. (B) After three years of recurrence, the sacral lesion grew to approximately 6 cm. (C) Following stereotactic ablative radiotherapy (SABR), the sacral lesion reduced in size after one year, allowing for a decrease in pain medication. (D) Two years post-SABR, the sacral lesion further reduced in size, and the patient no longer required medication for sacral pain management.

He had a medical history of thymectomy for Masaoka stage III thymoma, confirmed by pathological examinations, indicating direct pericardial invasion seven years ago. Five years prior, the patient presented with pain in the posterior aspect of the thigh. Computed tomography (CT) detected a 3-cm mass in the sacral bone (Figure 1A).

The lesion was biopsied, and immunohistochemistry showed AE1/3(+), p63(+), CK7(-), CK19(+), CK20(-), CD3(-), CD20(-), and TdT(-). The Ki-67 positivity rate was approximately 15%. Therefore, pathological examination indicated a metastatic thymoma involving the sacral bone. And, a 18F-fluorodeoxyglucose positron emission tomography scan was performed, confirming that this was the only site of recurrence.

The patient was started on denosumab and was prescribed chewable combination tablets containing calcium, natural vitamin D3, and magnesium. Following a multidisciplinary conference, surgery was deemed appropriate and recommended to the patient; however, the patient declined. Consequently, follow-up imaging revealed a tendency for the tumor to increase in size. Denosumab (120 mg) was administered subcutaneously every four weeks for three years. However, treatment was discontinued because of jaw osteonecrosis. By the time, he was referred to our department, the tumor had gradually grown to approximately 6 cm in size (Figure 1B).

**Figure 2:**
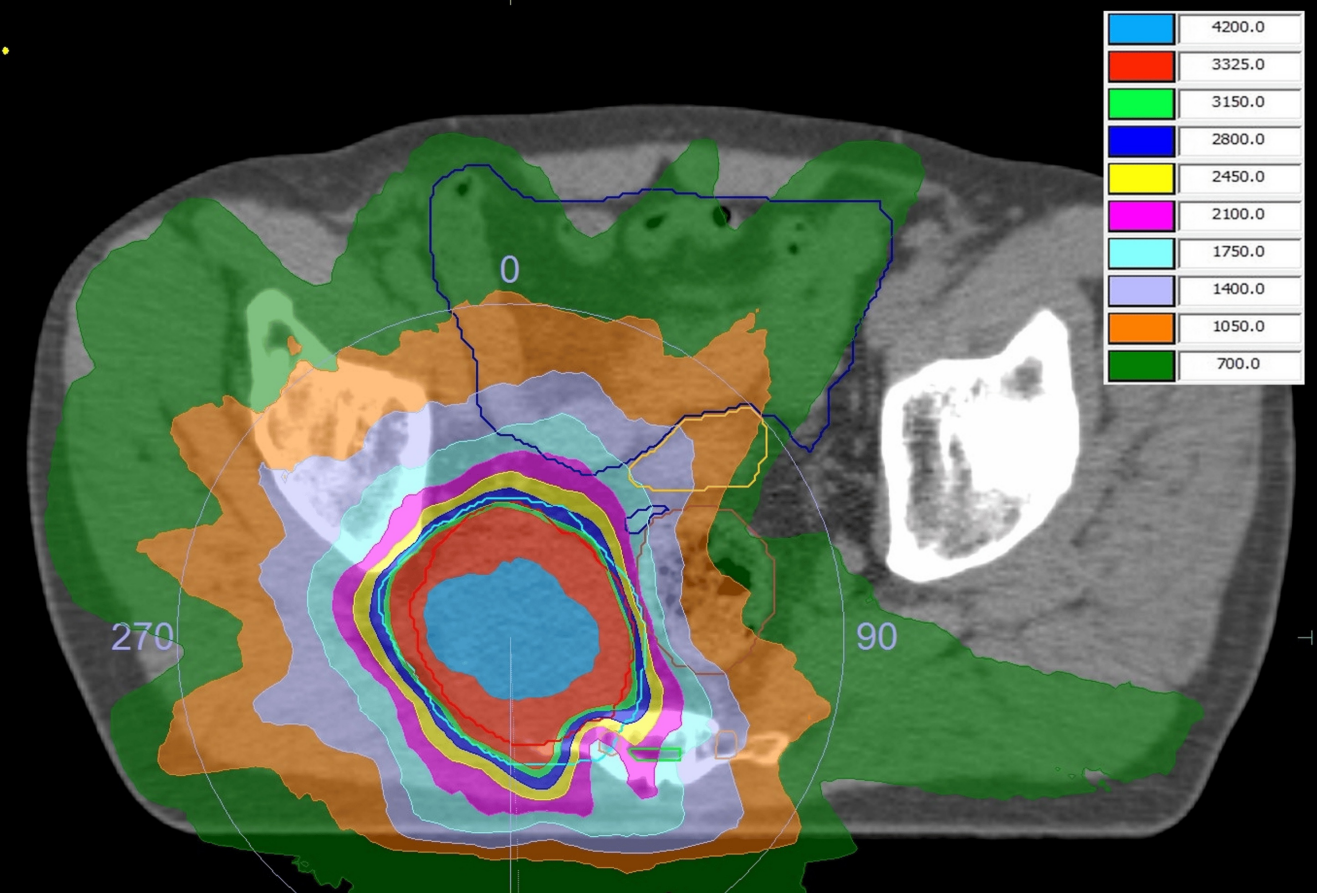
Dose distribution of stereotactic ablative radiotherapy Dose distribution of stereotactic ablative radiotherapy in the present case. The thin yellow line indicates the bladder and the thin blue line indicates the small bowel. The units for the numbers shown in the upper right label is centigray, which is one-hundredth (1%) of a gray.

In our department, SABR using volumetric modulated arc therapy is administered to sacral lesions. The prescribed total dose was 35 Gy in five fractions for the lesions (Figure 2).

The gross tumor volume (GTV) was defined as the volume of the contrast-enhancing lesion on CT. The clinical target volume (CTV) was equal to the GTV. The planning target volume (PTV) was created by adding an isotropic margin of 3 mm to the CTV in all directions. The treatment was planned such that the prescribed dose included 80% of the PTV. The remaining planning parameters are listed in Table 1.

**Table 1:** Dosimetric parameters for SABR in the present case Dosimetric parameters for SABR in the present case. PTV: Planning Target Volume. Vxx: the volume of the specific organ receiving over xx Gy. Dxx%: indicates the prescribed dose that includes xx% of the target volume.

Region of interest	Parameter	Value for the present case
PTV	Volume	216.736 cc
PTV	Min (D99%)	24.194 Gy
PTV	Max	47.399 Gy
Small bowel	Max	28.486 Gy
Small bowel	V19.5Gy	7.774 cc
Sacral plexus	Max	29.409 Gy
Sacral plexus	V30Gy	0 cc
Cauda equina	Max	28.585 Gy
Cauda equina	V30Gy	0 cc
Rectum	Max	24.993 Gy
Rectum	Mean	10.857 Gy
Bladder	Max	23.994 Gy
Bladder	Mean	9.116 Gy

The sacral lesion gradually decreased in size, and the pain medication was reduced after one year of SRT (Figure 1C). After two years, no medication was required for sacral pain management (Figure 1D).

## Discussion

Surgery is the preferred therapeutic option for potentially resectable recurrent thymomas as salvage therapy. Sandri et al. reported that rewarding long-term survival may be expected after treatment, especially when re-resection (radical) is performed, with a 82.4% survival rate of five years [9]. Marulli et al. also reviewed the outcomes and prognostic factors for 73 patients with recurrent thymoma treated surgically and found that complete macroscopic resection and single recurrence were associated with a significantly better prognosis, compared with incomplete resection and non-surgical treatment [10].

This case highlights the potential of SABR as a non-invasive treatment option for patients who are either not candidates for surgery or decline surgical intervention for resectable recurrent thymomas. Radiotherapy is used as adjuvant therapy after surgical resection or in a non-curative setting. We previously reported definitive hypofractionated radiotherapy as a curative treatment for locally advanced type B2 thymoma [11]. This case report provides new insights into thymoma management using SABR, especially given the limited number of studies on its use in settings with curative intent. Studies about stereotactic radiation therapy for patients with thymomas who are not candidates for surgery are limited. Pasquini et al. conducted a study involving 22 patients treated with SABR for pleural metastases [12]. They demonstrated a disease control rate of 79% after one year and 41% after two years, with a median progression-free survival of 20.4 months and no grade 3-4 toxicities. In another single-institution prospective study of 32 patients (39 target lesions) treated between 2005 and 2014, the response rate was 96.9%, with a median tumor shrinkage rate of 62.2% and a local control rate of 81.25% [13].

In the present case, we treated a single distant metastasis of the thymoma, classified as oligometastasis. Distant thymoma metastases are relatively uncommon. According to the International Thymic Malignancy Interest Group classification, recurrence is observed in approximately 11% of cases [14]. Studies have reported a significant difference in the survival outcomes between patients with single and multiple recurrences, with a 5-year overall survival rate of 86.2% for single recurrence and 61.3% for multiple recurrences (p = 0.005) [15]. In this specific case, it was concluded that radical therapeutic intervention was crucial for clinical outcomes.

## Conclusions

The optimal management strategy for recurrent thymomas remains controversial. Surgical resection of recurrent thymoma is the most commonly used approach for salvage therapy. However, in our case, the successful treatment of a solitary sacral recurrence of Masaoka stage III type A thymoma with SABR demonstrated the potential of this modality to achieve local control in rare and challenging clinical scenarios.

This case highlights the need for a multidisciplinary approach and individualized treatment planning in the management of thymoma recurrence. Ongoing follow-ups and further research are essential to validate the long-term efficacy and safety of SABR in similar cases and to refine therapeutic strategies for recurrent thymoma.
